# The Montreal Veterinary Medical Association

**Published:** 1893-03

**Authors:** 


*Montreal Veterinary Medical Association.*—The regular fortnightly meeting of Montreal Vet. Med. Association was held at the usual time and place (8 p. m., 6 and 8 Union Avenue, Feb. 10th, 1893. Dr. D. McEachran in the chair. There were also present: Drs. Mills, Morrow and Baker, and a large attendance of the members.

The usual routine of business having been disposed of, the Chairman called on Dr. Mills for a paper on the "Care and Breeding of Pigeons and Poultry and their Treatment in Disease."

This discourse was very instructive, and included much of the experience of the essayist as a breeder-exhibitor.

Considerable discussion resulted and the members, availing themselves of the rare privilege of discussing this subject, obtained much valuable information with regard to the various and almost unknown ailments of poultry and feathered pets.

Chicken cholera, gapes, roupe, etc., were pretty fully dealt with, while many of the minor ailments, such as impaction, tumors, etc., received due consideration.

Mr. Wilfred Plaskett reported a case of tetanus which he had treated successfully during the holidays.

The animal in question was a light bay mare, which had picked up a nail on the street about the 20th of July and became extremely lame. She was taken to a farrier and the nail was found to have penetrated the aufero-posterior part of the frog, and was removed and the lameness ceased. The nail, however, had penetrated the sensitive frog, and at the end of two weeks the animal refused food and showed a disinclination to move.

On further examination of the frog it was found that the whole of the horny frog had separated from the sensitive and had to be removed. The wound was dressed with medicated tar and opium, the animal placed in slings and box darkened so that she would be disturbed as little as possible.

*Symptoms were marked.*—Nose protruding, nostrils dilated, corners of mouth retracted, eyes sunken, membrana metotans reflected, profuse ensalivation, general muscular system in a state of tonicity, shown by method of standing; on manipulation, muscles were found to be inordinately hard, acceleration of breathing and other diagnostic features so well known and invariably constant in this condition. Temperature never showed a higher reading than 101 degrees, Fah., which was present on Aug. 4th. Pulse was not much altered, in fact symptoms pointed to a mild nature of the disease, there being an absence of hyperæsthesia, which marks the worse forms of tetanus.

*Treatment.*—Diet nutritious and in a fluid condition, so as to permit patient to suck up and swallow without attempting to masticate; oatmeal gruel and sloppy brans were constantly before her, in which there were placed her medicines, consisting of pot. brom., chloral hydrate and magnesium sulph. The two former were used for their motor depressant action; the latter to keep the bowels open. For the first ten days, after being placed in slings, she appeared very little different, except the excitement of being in slings caused a rise in temperature and pulse; of little consequence, however. After ten days a dccided improvement was observed. Jaws could be moved pretty freely.

At the end of three weeks process of mastication was pretty good, as the food no longer accumulated between the molars. She was taken out of the slings and groomed and any spots chafed were dressed. At the end of another week she was taken out, exercised, and, as she showed no signs of muscular rigidity, was pronounced cured.

The President asked Mr. Plaskett "on what physiological basis he prescribed such treatment."

Ans. The disease is of such a nature that it cannot be aborted, hence darkness, quietness, etc., all required to bring about a good result.

Ques. Explain quieting treatment.

Ans. The system when in this condition is more susceptible to stimuli than usual, consequently necessary to bring patient into an isolation from any noises, etc., and to overcome this hyperæsthesia to a certain extent with motor depressants.

Ques. What is tetanus due to?

Ans. A microbe which gets into a wound and whose toxines produce the tetanic spasms of the muscles. The microbe is supposed to remain at the wound and when the toxines attain a certain degree of virulence that the microbe is in turn killed.

The essayist of the evening, Mr. Stirrock, was then called upon by the chairman for his paper on navicular disease.

He commenced by stating that there was much conflicting opinion with regard to the different causes of this disease and the part of the navicular apparatus where these changes first began.

A short history of the disease was then given and the symptoms, progress and treatment of the disease fully dealt with it. As to the symptoms, they were very characteristic, and the pointing of the diseased limb one of the surest indications.

The predisposing causes were principally congenital, and the necessity of careful selection of animals in which there was an absolute freedom from any lack in conformation of the navicular apparatus, for breeding purposes, was demonstated. Exciting causes were chiefly traumatic ones. Bad shoeing being one of the most futile, exciting causes was proven by many of the cases which had come under the essayist's notice.

As to the treatment the essayist ventured to say that shoeing was the most radical and safe method in the early stages.

At the conclusion the President complimented the essayist on the style and thoroughness with which he had dealt with his subject. Considerable discussion ensued among the members with regard to the advisability of performing neurotomy in all cases.

Several questions were asked the essayist on the subject of shoeing in navicular disease.

Ques. How should a shoe be made to relieve chronic lameness from navicular disease?

Ans. By modelling the new shoes after the old ones.

Ques. In acute cases is it a good idea to shoe with high heels?

Ans. Perfectly level shoes the best.

Ques. Is it safe to perform neurotomy in case of animal having a corn?

Ans. No; as reparative processes would cease, and that is what is required in case of a corn.

Ques. Which would be most advisable in early stages frog seton or neurotomy?

Ans. Frog seton in early stages and neurotomy in later stages.

